# Deep Learning Identifies Abnormal Promyelocytes in Peripheral Blood Based on Morphological Analysis

**DOI:** 10.3390/diagnostics16071039

**Published:** 2026-03-30

**Authors:** Gongchen Wang, Guangyu Xu, Yao An, Minghui Xu, Zimeng Li, Yuanwei Feng, Tingting Li, Siqi Li, Mengxin Li, Zhijian Yang, Chunyan Gao

**Affiliations:** 1 Department of Medical Laboratory Science and Technology, Harbin Medical University-Daqing, Daqing 163000, China; wangzhigongchen@163.com (G.W.);; 2 Engineering Technology Research Center for Precision Diagnosis and Treatment of Frigid Zone-Related Diseases in Heilongjiang Province, Daqing 163319, China

**Keywords:** acute promyelocytic leukemia, deep learning, convolutional neural network, peripheral blood smear

## Abstract

**Background/Objectives**: Acute promyelocytic leukemia (APL) is a high-risk subtype of acute myeloid leukemia and requires rapid diagnosis to avoid early mortality. Current clinical diagnostic and genetic tests are time-consuming, expensive, and complex. Notably, all these tests depend on bone marrow aspiration and are intensely invasive, resulting in poor patient compliance. This study aimed to develop a rapid, explainable, and accurate auxiliary tool for cell-level detection of abnormal promyelocytes in peripheral blood smears, which can serve as a key clue for suspecting APL. **Methods**: We developed a multi-stage deep learning (DL) model that automatically read images of peripheral blood smears (PBSs), accurately segmented cells, and identified abnormal promyelocytes using only image data. We retrospectively reviewed a total of 114 bone marrow smears (42 APL patients and 72 non-APL patients) and 158 PBSs (30 APL patients and 128 non-APL patients) at the Fifth Affiliated Hospital of Harbin Medical University and collected 223,123 cell images for training. Then, the efficacy of EfficientDet in APL screening was evaluated with an additional 150 PBSs (50 from APL patients and 100 from non-APL patients) and finally compared with manual microscopy. **Results**: EfficientDet exhibited superior overall screening performance compared with pathologists in the identification of abnormal promyelocytes. **Conclusions**: Our findings suggest that the DL approach we describe herein is promising as a practical tool for abnormal promyelocyte detection and early APL screening, raising attention to suspected cases of APL for expert evaluation and further reducing diagnostic delays.

## 1. Introduction

Acute promyelocytic leukemia (APL) is a distinct subclass of acute myeloid leukemia that is characterized by the excessive proliferation of atypical promyelocytes in bone marrow and peripheral blood [1]. It has been considered one of the deadliest leukemias, and the mortality among Chinese urban residents increased from 3.60/100,000 in 1991 to 3.72/100,000 in 2015, which seriously endangers human health [2]. Although the prognosis of APL patients has been dramatically improved with the advent of all-trans retinoic acid and arsenic trioxide, their early mortality rate remains alarmingly high at 32% [3,4,5]. Most importantly, as a hematologic emergency, APL has an insidious onset, rapid progression, and serious consequences [6]. Therefore, timely and accurate diagnosis is important to enable efficient treatment and improve the prognosis of patients with APL. At present, definitive diagnosis of APL requires molecular confirmation of PML-RARA translocation, but these techniques are time-consuming and unavailable in many countries with limited healthcare resources. Bone marrow and peripheral blood smears (PBSs) for pathological examination are considered to be the primary standard in the diagnosis of APL. However, this approach is time-consuming and labor-intensive, and requires well-trained technicians [7,8,9]. Consequently, it is vital to find an efficient, low-cost, and easily operable subsidiary diagnosis method.

Artificial intelligence and its branches have shown excellent capabilities in medical image interpretation tasks, which can be applied to computer vision for blood cell morphology differentiation [10,11]. Convolutional neural network (CNN), as the latest core model of deep learning (DL), has outstanding advantages in image recognition and feature extraction because it can imitate the natural visual processing of the brain and can interpret dense information [12,13]. The classification of physiological cell types in PBS and the distinction between malignant cells and healthy cells have been successfully accomplished by a variety of CNN models [14,15,16]. However, a critical limitation of previous studies is that they focused on a relatively small number of disease classes using DL approaches to blood cell classification. Most importantly, few attempts have been made to diagnose and typify leukemia by blasts that show up in PBS, particularly for APL screening and diagnosis [17,18,19].

In this study, we presented a highly sensitive and economical AI-based method for the morphological identification of promyelocytes. This novel approach could effectively improve patient compliance and enable faster screening for APL for early diagnosis and treatment.

## 2. Materials and Methods

### 2.1. Patient and Sample Collection

This retrospective study included a cohort of 422 patients admitted to the Fifth Affiliated Hospital of Harbin Medical University between September 2020 and September 2025 and collected their PBSs or bone marrow smears. All the smears were digitized and annotated. The cohort consisted of 122 patients diagnosed with APL and 300 patients with non-APL smears. The non-APL cohort specifically comprised the following diseases and populations: severe pneumonia, myelodysplastic syndrome, primary myelofibrosis, megaloblastic anemia, rheumatoid arthritis, and healthy controls. The clinical and biological characteristics of the patients are detailed in Table 1.

The 422 patients constituted the full study cohort, which was further divided into mutually exclusive training and independent validation sets without any overlapping cases. Specifically, 272 patients (corresponding to 114 bone marrow smears and 158 PBSs) were allocated to the model training set, while the remaining 150 patients (corresponding to 150 PBSs) were utilized for independent validation of the model.

In this study, all the smears were stained using a Wright–Giemsa staining kit (BASO Co., Ltd., Zhuhai, China). APL cases were diagnosed in accordance with the 2022 World Health Organization classification of tumors of hematopoietic and lymphoid tissues, as well as the International Consensus Classification criteria, with laboratory tests performed in accordance with the standards recommended by the International Committee for Standardization in Hematology. The diagnosis was confirmed by the presence of the PML-RARA fusion gene (detected via RT-PCR or fluorescence in situ hybridization), and risk stratification was performed using the 2022 European Leukemia Net risk classification system for APL.

### 2.2. Digitization and Data Preparation

All the smears were converted into digital images using the Aperio VERSA 8 Scanning System (Leica, Wetzlar, Germany), a fully automated digital pathology scanner, resulting in Whole Slide Images (WSIs) with a pixel size of 0.5 µm. It was worth noting that the middle area with appropriate smear thickness, uniform cell distribution, and good staining was the best area for model recognition, while the starting and ending ends were not suitable for recognition and observation. Meanwhile, the cell numbers obtained from each smear varied because of variations in smear quality, typically ranging from 400 to 1500 nucleated cells per smear.

### 2.3. Image Segmentation and Recognition Model

Following the digitization of all the smears, we used the U-Net and EfficientDet models for segmentation and recognition tasks. The U-Net model was adopted to complete the key steps of cell image segmentation. Quantitative evaluation of the segmentation model was conducted using the Dice coefficient and mean intersection over union (mIoU). Raw confusion matrix counts, including the true positives, false positives, true negatives, and false negatives, were calculated for each test sample. Meanwhile, U-Net was applied to segment 114 bone marrow smears and 158 PBSs, generating a total of 223,123 single-cell images. These images were standardized to a uniform size of 512 × 512 pixels while retaining background components such as erythrocytes, platelets and cell fragments. EfficientDet was responsible for the subsequent cell classification and recognition tasks, and the above 223,123 U-Net-segmented and standardized single-cell images were partitioned into a training set (80%) and a test set (20%) for the training and performance validation of the EfficientDet model, respectively. The training dataset comprised two parts: the APL set and the non-APL set. Notably, there were approximately 2.5 times more non-APL images than APL, which led to an imbalance problem during training. To address this issue, we adopted a balanced class weight strategy during model training, which helped achieve a more balanced performance between sensitivity and specificity. Furthermore, an independent validation set was constructed from 23,986 single-cell images extracted from 150 PBSs via the identical U-Net segmentation algorithm to further evaluate the clinical applicability of the EfficientDet model.

### 2.4. Model Training and Implementation Details

To ensure the reproducibility and comparability of all the experiments, the critical hyperparameters and training strategies for the U-Net segmentation model and EfficientDet were strictly controlled and quantitatively documented. The detailed key training settings, including learning rate, optimization algorithm, batch size, and early stopping criteria, are listed in Supplementary Table S1. In addition, the key training and implementation details of EfficientDet include specific EfficientDet variants/configurations, input resolution, loss function, augmentation strategy, class-imbalance handling, transfer learning details, and the method used to select the confidence threshold, all of which are shown in Supplementary Table S2.

### 2.5. Using the Strategy of Transfer Learning

EfficientNet was used as the feature extraction model to learn the image features automatically from the preprocessed digitized cell images. It collaborated with the MBConv module through a composite scaling strategy to extract multi-scale cell features and integrate hierarchical semantics. Based on the preset confidence threshold, it initially completed the cell classification prediction and then inputted the optimized cell features into the linear classification network to achieve precise classification.

**Figure 1:**
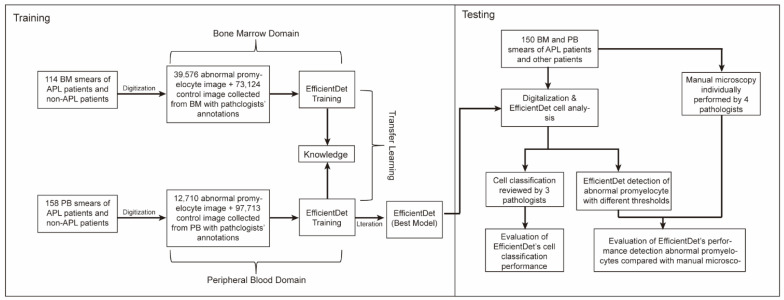
Flow chart design of the study.

To improve the generalization ability of the model and prevent overfitting, the EfficientDet model requires a large amount of sample data. However, given the rarity of abnormal promyelocytes in peripheral blood, it is difficult to collect sufficient data for EfficientDet. To solve this problem, a transfer learning approach was implemented. A smaller cell dataset from PBS was used to fine-tune EfficientDet specifically for the detection of abnormal promyelocytes after it had been trained on a large-scale cell dataset from bone marrow smears. Indeed, knowledge from a broader dataset was transferred, only optimizing the final classification layer to facilitate efficient learning and adaptation to the target task, as shown in Figure 1.

### 2.6. Gradient-Weighted Class Activation Mapping (Grad-CAM)

Grad-CAM (1.4.8) is a tool used for visual explanations of CNNs [20]. It uses class-specific gradient information flowing into the final layer of a CNN to produce a map of important regions in the image. The last convolutional layer is the layer immediately before the final layer that generates class predictions. Consequently, the neurons in this last convolutional layer should summarize which features in the image are important in making these predictions. Grad-CAM produces heatmaps that, when overlaid on the original image, correspond to the image regions that were significant for predicting the output class.

### 2.7. Performance Evaluation

We invited four pathologists with more than 5 years of experience to review the cell recognition results and assess the performance of the EfficientDet model in detecting abnormal promyelocytes. Each case was independently reviewed by three pathologists. The cell interpretation was considered valid when three pathologists agreed on the classification of abnormal promyelocytes. If the classification was inconsistent, a fourth pathologist was consulted as an arbiter. These reviewed cell classifications were then compared with those generated by EfficientDet. Finally, sensitivity, specificity, and accuracy were evaluated based on the abilities of EfficientDet and the individual pathologists to classify cells (Table 2).

### 2.8. Statistical and Data Analysis

All the models were built in Python version 3.8.20 with Keras version 2.9.0 and TensorFlow version 2.9.1. Computations were performed using a high-performance computing system equipped with an AMD Ryzen 9 7960X processor and an NVIDIA GeForce RTX 4090 GPU. All statistical analyses were performed using GraphPad Prism 9 (GraphPad Software Inc., San Diego, CA, USA). Quantitative results are presented as the mean with 95% confidence intervals (95% CI). Statistical significance was assessed using the Mann–Whitney U test, with *p* < 0.05 considered statistically significant.

## 3. Results

### 3.1. Pretreatment and Identification of PBS and Bone Marrow Smear

**Figure 2:**
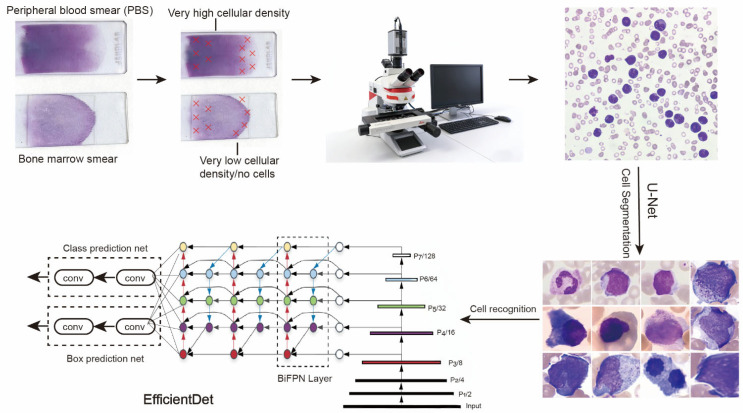
Workflow of EfficientDet to classify and recognize smears.

We collected data from 422 patients with and without APL and found that the median age was mainly consistent between the APL and non-APL cohorts (with an overall range of 50–75 years). Table 1 provides information on the characteristics of the patients. To digitize PBSs and bone marrow smears from these patients, we first filtered the areas with uneven cell density and blurred images throughout the entire smear. Full-field scanning of the remaining area of the smear was performed using the Aperio VERSA 8 Scanning System to generate a WSI. Then, the WSI was input into the U-Net model to generate single-cell images according to the cell profile. Finally, each cell image was characterized by the various sizes, shapes, and color variations in the nuclei and cytosol, as well as annotated for visualization using the EfficientDet model (Figure 2).

### 3.2. Identification of Control White Blood Cells and Abnormal Promyelocytes in Peripheral Blood

**Figure 3:**
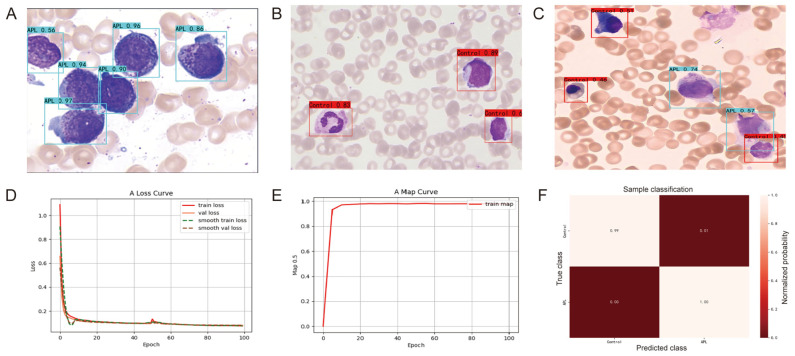
EfficientDet recognizing control and abnormal promyelocytes in smears. Recognizing (**A**) the abnormal promyelocytes from APL patients’ smears and (**B**) the control cells from non-APL patients’ smears. (**C**) Recognizing the abnormal promyelocytes and control cells from APL patients’ smears. (**D**) A loss curve. (**E**) A map curve. (**F**) The confusion matrix of the classification model.

The first step in constructing the EfficientDet model was data preprocessing to normalize data using TensorFlow (2.9.1), NumPy (1.24.4), OpenCV (4.12.0.88), Pandas (2.0.3), and Matplotlib (3.7.5) packages, along with activation functions including Swish, ReLU, Sigmoid, and Softmax. Next, we trained and tested our EfficientDet model to predict diagnostic labels from image fields of all the samples. To verify the validity and reliability of the EfficientDet model, we tested it using the training and testing datasets and applied multiple validation methods. The testing step confirmed that the model could predict data not used in training, indicating the high diagnostic accuracy of the EfficientDet model. Finally, we used the model to predict images from APL patients and non-APL patients (Figure 3A–C), which validated the accuracy of the classification model. Meanwhile, we also examined the loss rates (<0.05 ± 0.01) and mean average precision (≥0.98 ± 0.01) of this model (Figure 3D,E), which showed that the EfficientDet model was able to recognize the control cells and the abnormal promyelocytes with high accuracy. In addition, the cross-validation yielded an average accuracy for both classes: 0.99 ± 0.01 (Figure 3F). However, the datasets used to train the EfficientDet model were single-cell level annotations, and the recognition accuracy for multicellular image classification was not high.

### 3.3. Evaluation of Segmentation Performance of U-Net Segmentation Model

**Figure 4:**
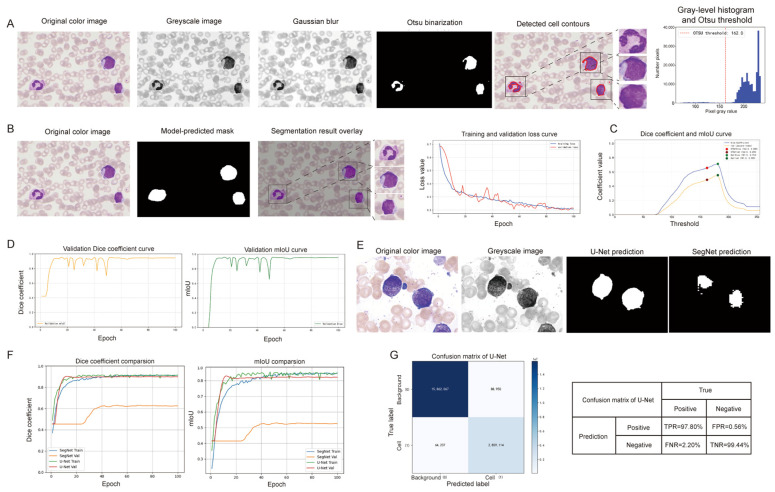
The contrast of the segmentation models: (**A**) The performance of cell segmentation of the Otsu model. (**B**) The performance of cell segmentation of the U-Net model. (**C**) The Dice coefficient and mIoU curves of the Otsu model. (**D**) The validation Dice coefficient and mIoU curves of the U-Net model. (**E**) The cell segmentation results of U-Net and SegNet on the same sample. (**F**) The comparison of the Dice coefficient and mIoU between the SegNet and U-Net models. (**G**) Confusion matrix of U-Net.

The most challenging task in blood cell analysis is image segmentation, because of the complexity of cell structure and the overlaps of cells. In APL diagnosis, accurate image segmentation plays an important role, as it is used for the extraction of all overlapping objects present in an image. Thus, the accuracy of the classification and recognition of abnormal promyelocytes depends on the accurate segmentation of PBS. The Otsu method, which is one of the most popular automatic segmentation approaches for grayscale images, is an adaptive threshold segmentation algorithm for gray-level image histograms. Meanwhile, it can automatically select the optimal threshold by maximizing the between-class variance in gray levels between an object and background, resulting in the automatic separation of the target and background in PBS. In this study, we obtained a series of single-cell images using the Otsu model (Figure 4A). However, its adaptability to uneven illumination and complicated overlapping cell scenarios is poor. In addition, the U-Net model is a segmentation model based on a CNN and concatenates the contraction and expansion paths through skip connections. Its contraction path uses 3 × 3 no-padding convolution, ReLU activation, and 2 × 2 maximum pooling to achieve the doubling of down-sampling and feature channels; in the expansion path, upsampling is performed via transposed convolution, a high-resolution feature from the encoder is fused via skip connections, and a 1 × 1 convolution is applied to obtain the final segmentation masks. Its segmented single-cell images have clear cell contours and boundaries, which can retain more local details of primary cells, such as the depressed borders of the nuclei. They have morphological uniformity between the segmented single cells and primary cells (Figure 4B). In addition, we further utilized Intersection over Union (IoU) and the Dice coefficient for quantitative evaluation to objectively compare the segmentation performances of the U-Net and Otsu methods. The results showed that U-Net achieves optimal results in IoU and Dice, indicating that the single-cell segmentation of U-Net is more accurate and consistent (Figure 4C,D).

To further evaluate the segmentation performance of U-Net, we compared it with another image segmentation model based on a CNN, namely SegNet. As shown in Figure 4E, the cell contours generated by U-Net are more complete and smoother, and highly match the real situation, while SegNet exhibits fragmented boundaries and missing regions. In addition, on the validation set, U-Net achieved a Dice of 0.91 (95% CI: 0.90–0.92) and mIoU of 0.83 (95% CI: 0.82–0.84), significantly outperforming SegNet, which yielded a Dice coefficient of 0.63 (95% CI: 0.62–0.64) and mIoU of 0.52 (95% CI: 0.51–0.53). The performance gap between the training and validation curves of U-Net was far smaller than that of SegNet, demonstrating superior model robustness and generalization ability (Figure 4F). We further present the confusion matrix of the U-Net segmentation model. It achieved high true positive (97.80%) and true negative (99.44%) rates, with minimal false predictions, consistent with the superior Dice and mIoU performance observed in the validation curves (Figure 4G). Thus, single-cell images are more accurately obtained using the U-Net segmentation model.

### 3.4. Single-Cell Image Recognition and Assessment of Pathologists

**Figure 5:**
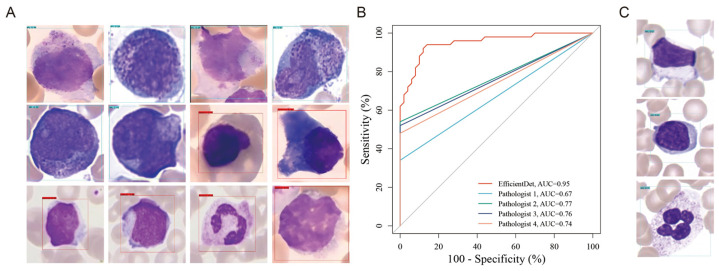
Single-cell image recognition and assessment by the pathologists: (**A**) The results of recognition of the single-cell images. (**B**) Receiver operating characteristic (ROC) curves with the AUC noted. (**C**) Representative false-positive misclassification images.

In this study, we used the U-Net model to obtain single-cell images, then the cell images were sorted and identified using the EfficientDet model. We found that the accuracy of single-cell image recognition and classification was higher than that of whole-image recognition and classification, with an increase of 30–40% (Figure 5A). Subsequently, the performance of EfficientDet in identifying abnormal promyelocytes was compared with the assessment results of four independent pathologists using the ROC curves. The area under the ROC curve (AUC) measures detection performance, with a value closer to one indicating superior performance. The results showed that the AUC values of the four pathologists were 0.7963, 0.8065, 0.7841, and 0.7954, which were significantly lower than those of EfficientDet (0.9426) (Figure 5B). Moreover, the performance of EfficientDet in detecting abnormal promyelocytes was further quantitatively assessed and compared with that of the pathologists, particularly in sensitivity, specificity, accuracy, and F1 score. We found that the sensitivity of EfficientDet was 0.97, approximately double that achieved by the pathologists, while maintaining a specificity of 0.85. The accuracy of EfficientDet was 0.96, which was similar to that of the pathologists. Notably, the F1 score of EfficientDet reached 0.98, far exceeding that of all four pathologists, reflecting its superior comprehensive classification performance (Table 2). However, the specificity of EfficientDet was lower than that of the pathologists. Visual analysis of false-positive cases revealed normal leukocytes misclassified as abnormal promyelocytes, driven by cellular artifacts (staining artifacts or edge blurring) and image noise (Figure 5C). Clinically, such false-positive errors may lead to false-positive APL screening outcomes, resulting in unnecessary further examinations and causing unnecessary psychological stress to patients. These findings highlight the need to optimize specificity via expanded training on diverse normal cell phenotypes.

### 3.5. Grad-CAM Heatmaps of EfficientDet

**Figure 6:**
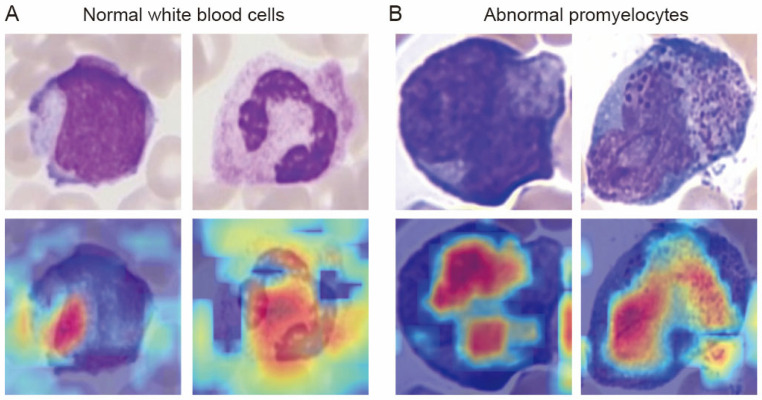
Grad-CAM attention maps of EfficientDet. Original images of (**A**) normal white blood cells and (**B**) abnormal promyelocytes. Bottom: Grad-CAM heatmaps showing model focus regions.

To validate the interpretability of EfficientDet and enhance the credibility of its decision-making process, we generated class activation maps using a Grad-CAM approach to represent the regions of interest that the model focused on during cell classification. For normal white blood cells, the EfficientDet focused on the segmented nuclear regions and cytoplasmic boundaries. In contrast, for abnormal promyelocytes, the EfficientDet showed strong activation in the granular cytoplasm and at the contours of the twisted nuclei, which are precisely the core diagnostic markers of APL (Figure 6A,B). These results demonstrated that our model identifies biologically relevant features rather than spurious patterns.

## 4. Discussion

Definitive diagnosis of APL requires morphological analysis, flow cytometry analysis, molecular genetics, and confirmation of chromosome t (15;17) or the PML::RARA fusion gene, all of which are time-consuming and not applicable in underdeveloped medical regions. As a hematologic emergency, APL requires immediate treatment upon suspected diagnosis, both causal and supportive, because of its rapid progression and life-threatening coagulopathy [21,22,23]. Therefore, it is imperative to develop simple and highly effective methods for screening and early diagnosis of APL. With the rapid development of AI-assisted morphology analysis, it has become a hot spot in medical research and is widely used for cell identification, distinction, and classification [24,25,26,27,28]. Here, we present a DL-based system designed to rapidly detect and differentiate abnormal promyelocytes in PBS to support the diagnosis of APL, which could promote the prioritization of APL in currently overloaded healthcare systems and play a vital role in low-resource settings.

The existence of abnormal promyelocytes with characteristic bundled Auer bodies in PBS is considered a diagnostic criterion for presumptive APL diagnosis. In a computer-aided diagnosis framework for evaluating cancer cell morphology based on a CNN, accurate cell segmentation is a key initial step [29,30]. Traditional CNNs have a limitation in that they require a sizable image cohort to ensure their effectiveness, while U-Net networks have been shown to have the ability to segment biomedical images effectively with minimal training data [31,32,33]. In addition, traditional digital image-processing algorithms, such as the Otsu algorithm, are also widely used in image segmentation techniques for their advantages of convenience and stability [34]. In order to ensure the validity of the model under the limited sample size, we compared the segmentation performance of the U-Net and the Otsu algorithm. The results showed that compared with the Otsu algorithm, regions segmented by U-Net are highly consistent with the boundaries of abnormal promyelocytes, possess significantly superior cell region integrity and edge accuracy, and more accurately cover the actual scope of target cells. Thus, we applied the U-Net segmentation model to this study.

What is different from previous studies is that we assist in diagnosing diseases based on cell morphology, rather than just classifying the cells. We evaluated the efficacy of our EfficientDet model in APL recognition and found that it achieved high sensitivity, specificity, and accuracy. The time required to upload the PBS image to the model and then output the results was 12.5 s. Most importantly, compared with the independent microscopic evaluation results of pathologists, the sensitivity and accuracy of the EfficientDet model in detecting abnormal promyelocytes were significantly superior to those of pathologists, highlighting its high potential clinical application value. Indeed, the automated approach of EfficientDet rapidly evaluated numerous cells in PBS, identifying those that might be abnormal promyelocytes, flagging them for immediate evaluation by hematopathologists, and enabling efficient APL screening. Therefore, the EfficientDet model is expected to serve as a rapid preliminary screening tool for APL, streamlining diagnostic workflows in clinical practice.

### Limitations of the Study

There are several limitations of the current work that need to be improved in the future. First, the sample size of our study was relatively small due to the low prevalence of APL, and larger sample size studies may be needed in the future. Second, the current work lacks external validation using images from open-source datasets or an external test cohort from an independent center, which raises the risk that the model may be overfit to the hospital-specific staining protocols. Third, this study fails to perform prospective validation of real-life efficacy in improving the diagnostic performance of clinicians (comparing clinicians with AI vs. clinicians alone). Fourth, our model only considers cytomorphology and has not integrated key clinical information such as routine blood test results. Combining clinical information with cell morphology images through a multimodal framework or visual language models will help improve diagnostic accuracy and provide an even stronger decision support system for clinicians. Furthermore, a recent study indicated that vision transformers excel in capturing global context and refining lesion boundaries in medical images, whereas YOLO-based architectures are favored for their lightweight and efficient inference [35]. These next-generation vision architectures show great potential in medical image analysis. However, systematic comparisons between EfficientDet and these advanced architectures were not performed in the present study.

## Figures and Tables

**Table 1:** Characteristics of patients.

Parameter	Non-APL	APL
*N*	300	122
Age (year)	60 ± 10	65 ± 10
Male (%)	47.3	46.8
Female (%)	52.7	53.2
WBC (×10^9^/L)	30.1 ± 15.2	3.4 ± 1.5
HGB (g/L)	95.0 ± 15.2	100.2 ± 15.4
PLT (×10^9^/L)	96.6 ± 30.3	35.0 ± 20.8
PB abnormal promyelocytes	/	40.2 ± 20.7
BM abnormal promyelocytes	/	60.9 ± 14.5

Abbreviations: WBC, white blood cell; HGB, hemoglobin; PLT, platelet; PB, peripheral blood; BM, bone marrow.

**Table 2:** The sensitivity, specificity, and accuracy of four pathologists and EfficientDet for abnormal promyelocyte detection.

Pathologists	Sensitivity	Specificity	Accuracy	F1 Score
Pathologist 1	0.4532	1.0000	0.9563	0.6237
Pathologist 2	0.5267	1.0000	0.9673	0.6900
Pathologist 3	0.5087	0.9956	0.9245	0.6630
Pathologist 4	0.4853	0.9985	0.9468	0.6476
Recognition Model EfficientDet	0.9684	0.8506	0.9589	0.9775

## Data Availability

All images and raw data for statistical analysis in this study are available from the corresponding author upon reasonable request.

## Supplementary Materials

The following supporting information can be downloaded at https://www.mdpi.com/article/10.3390/diagnostics16071039/s1, Table S1. Training hyperparameters for U-Net and EfficientDet; Table S2. Key training and implementation details of EfficientDet.
