# Supplementary material for: Deep Learning Identifies Abnormal Promyelocytes in Peripheral Blood Based on Morphological Analysis
Source: Diagnostics (Basel). 2026 Mar 30;16(7):1039. doi: 10.3390/diagnostics16071039 (PMC13073449; doi:10.3390/diagnostics16071039)
Supplement: Supplementary file 1 [file diagnostics-16-01039-s001.zip › diagnostics-4142947-supplementary.pdf]

Table S1. Training hyperparameters for U-Net and EfficientDet.

Hyperparameter Category	U-Net	EfficientDet
Initial learning rate	1×10^{-4}	3×10^{-4}
Optimization algorithm	Adam	Adam
Batch size	8	8
Early stopping criteria	monitored index: validation loss; minimum change threshold: 0; patience: 15 epochs	monitored index: validation loss; minimum change threshold: 0; patience: 15 epochs

Table S2. Key training and implementation details of EfficientDet.

Parameter Category	Detailed configuration
EfficientDet variants and core architecture	EfficientDet-D0 (phi=0); backbone: EfficientNet-B0; feature fusion: BiFPN; anchor scales: [32, 64, 128, 256, 512].
Input resolution	512×512
Loss function	Classification: focal loss (γ=2, α=0.25); regression: smooth L1 loss (β=1.0); loss weight ratio (classification: regression) = 1:1
Augmentation strategy	Training set only: random horizontal flip (p=0.5), random crop (0.8-1.0), random scale (0.5-1.5); brightness/contrast/saturation adjustment (±10%); mosaic augmentation
Class-imbalance handling	Balanced class weight strategy (based on inverse sample size ratio)
Transfer learning details	Pre-trained weights: VOC dataset-based EfficientDet-D0 weights (loaded by_name=True, skip_mismatch=True); frozen layers: first 226 layers (50 epochs); unfrozen all layers (100 epochs); unfrozen LR: scaled by batch_size/16×initial LR
Confidence threshold selection	Initial threshold: 0.5 (confidence), 0.5 (NMS); optimal threshold: grid search (0.45-0.55) on validation set for maximum F1-score
